# Massive lamotrigine and bupropion overdose resulting in status epilepticus without cardiovascular collapse

**DOI:** 10.1080/24734306.2019.1699750

**Published:** 2019-12-22

**Authors:** Rebecca E. Bruccoleri, Bradley L. Demeter, Peter R. Chai, Michele M. Burns

**Affiliations:** a Division of General Pediatrics, Boston Children’s Hospital, Boston, MA, USA;; b Emergency Medicine, Brigham and Women’s Hospital, Boston, MA, USA;; c Emergency Medicine, Beverly Hospital, Beverly, MA, USA;; d Emergency Medicine, Program in Medical Toxicology, Boston Children’s Hospital, Boston, MA, USA

**Keywords:** Lamotrigine, Bupropion, Case Report

## Abstract

An 18 year-old woman presented to an outside hospital with seizure activity after a massive ingestion of lamotrigine, bupropion, trazodone, buspirone, and possibly isoretinoin. Her initial vital signs were remarkable for tachycardia (120 bpm). She was intubated for airway protection. For treatment of status epilepticus, she received a total of 12 mg of IV lorazepam along with a lorazepam infusion titrated to 15 mg/hr, a propofol infusion of unknown dosing, and phenobarbital 650 mg. She was transferred to a receiving hospital. Her initial ECG at the receiving hospital showed a QRS of 117 ms which narrowed with 50 mEq of sodium bicarbonate after approximately 6 hours. She required norepinephrine intermittently for blood pressure support for approximately 2 days. The patient had no dysrhythmias. EEG showed no epileptiform activity from approximately 11 hours–32 hours post ingestion. At the receiving hospital, her serum lamotrigine concentration was 109 mcg/mL (reference 3.0–14.0 mcg/mL) 7 hours after ingestion. Her bupropion concentration was 92 ng/mL (reference 50–100 ng/mL). She was extubated on hospital day 5 and discharged to a psychiatric facility on hospital day 13.

## Introduction

Lamotrigine and bupropion are two medications prescribed to treat psychiatric disorders. Both are known to cause seizures and potentially fatal dysrhythmias in overdose. We describe a case of a massive lamotrigine, bupropion, trazodone, buspirone, and possibly isoretinoin ingestion that resulted in status epilepticus, QRS widening, and hypotension that responded to vasopressors without cardiovascular collapse. Her initial serum lamotrigine concentration was 109.0 mcg/mL which is the highest reported in the literature.

## Case description

An 18 year-old woman with reported history of three suicide attempts presented to an community hospital after an ingestion of unknown quantities of lamotrigine, bupropion, trazodone, buspirone, and possibly isoretinoin. However, by family history, she mostly likely took greater than 10 grams of lamotrigine. Her initial vital signs were temperature: 97.0 degrees F, heart rate: 120 beats per minute, blood pressure: 115/66 mmHg, respiratory rate: 16 breaths per minute, oxygen saturation: 94% with bag mask ventilation. Weight was estimated at 72.6 kg. She arrived obtunded but had a gag reflex and would withdraw to stimuli. Her pupils were equal in size, round, and sluggishly reactive to light. She was intubated for airway protection and decontaminated with activated charcoal *via* nasogastric tube. She then had over 30 min of clinical seizure activity consistent with status epilepticus as defined by the American Epilepsy Society [1]. Seizure activity when described was reported as tonic-clonic. She received a total of 12 mg of IV lorazepam, a lorazepam infusion titrated to 15 mg/hr, and a propofol infusion (30 mcg/kg/min at time of transport arrival). Given persistent seizure activity, she was given a total of phenobarbital 650 mg.

Her laboratory values were significant for sodium of 140 mEq/L, 3.2 mEq/L, chloride 104 mEq/L, carbon dioxide 23 mEq/L, BUN 7 mg/dL creatinine0.7 mg/dL, glucose 141 mg/dL, AST 22 units/L, ALT 17 units/L. Her salicylate, acetaminophen, and ethanol concentrations were undetectable. Her urine drug screen was negative for amphetamines, benzodiaze-pines, THC, cocaine, methadone, opiates, oxycodone, and buprenorphine. Her ECG at the initial hospital showed sinus tachycardia 155 bpm without QRS widening.

During transfer to a receiving hospital, propofol and lorazepam infusions were discontinued, and she received 1 mg of lorazepam and a total of 100 mcg of fentanyl. She also was started on norepinephrine en route for hypotension (79/47 mmHg) (the nadir of her BP in transport was 65/27 mmHg) and she responded well to norepinephrine.

On arrival to the receiving hospital, she had sluggishly reactive pupils, present corneal reflexes, absent gag reflex, hypotonia with no volitional movements, no response to painful stimulus, no hyperreflexia, and no clonus. Her initial ECG at the receiving hospital showed a sinus rhythm of 96 bpm with a QRS of 117 ms, QT 394 ms, and QTcB of 497 ms. She received 50 mEq of sodium bicarbonate for QRS prolongation greater than 100 ms, and her repeat QRS was 116 ms which narrowed to 102 ms approximately 6 h later without further intervention. She received 2 grams of magnesium for a magnesium concentration of 1.5 mg/dL. Her serum calcium was 7.6 mg/dL (ionized 1.06 mmol/L) and normalized without intervention. Her QTc normalization 2 days later which was not correlated with the normalization of magnesium or calcium. She required norepinephrine intermittently for blood pressure support (max dose 0.15 mcg/kg/min) for approximately 2 days after admission to the receiving center. The patient had no known dysrhythmias. Her EEG showed no epileptiform activity from approximately 11 h–32 h post ingestion.

At the receiving hospital, her serum lamotrigine concentration was 109 mcg/mL (reference 3.0–14.0 mcg/mL) approximately 7 h after ingestion. Her serum trazodone concentration was 2.2 mcg/mL (ref. 0.50–2.50 mcg/mL) and serum buspirone concentration was undetectable approximately 15 h after ingestion. Her serum bupropion concentration was 92 ng/mL (reference 50–100 ng/mL) approximately 20 h after ingestion. Subsequent lamotrigine concentrations declined to 10.2 mcg/mL and 2.5 mcg/mL at 3 and 6 days later, respectively. She was extubated on hospital day 5 and discharged to a psychiatric facility on hospital day 13.

## Discussion

This case described status epilepticus without cardiovascular collapse in the setting of mixed lamotrigine, bupropion, trazodone, buspirone, and possible isoretinoin overdose. Her buspirone concentration was undetectable but taken 15 h after ingestion and therefore, does not rule out the possibility of ingestion. Her serum lamotrigine concentration of 109.0 mcg/mL is the highest reported in the literature. Her serum bupropion concentration was in the reference range but drawn approximately 20 h after ingestion and therefore, has the possibility of being higher at time of presentation. The onset of seizures and subsequent status epilepticus without a herald event like cardiovascular collapse is notable and important for clinicians to recognize. There was limited information about her initial neurological exam and status epilepticus dominated the clinical picture. In the setting of drug induced seizures, it is important to follow a stepwise approach of escalating drug therapy for seizure control while monitoring for hemodynamic and ECG changes. This case demonstrates such an approach with endotracheal intubation, a high dose lorazepam infusion, propofol infusion, and phenobarbital. Her QRS widening narrowed with only 50 mEq of sodium bicarbonate over the course of approximately 6 h and therefore, it is unclear how effective sodium bicarbonate was in her QRS narrowing. She required norepinephrine but it is unclear if this was related to her ingestion (trazodone has alpha-1 antagonism) or significant doses of sedation/anticonvulsants required for seizure control.

Lamotrigine, bupropion, and trazodone all can cause seizures after overdose. There are limited reports of buspirone overdose, but seizures have been associated with a buspirone overdose in case report. However, in this case, the seizure occurred approximately 36 h after the overdose [2]. QRS prolongation can occur with both lamotrigine and bupropion which was seen in our patient. Her QRS prolongation was responsive to sodium bicarbonate. Bupropion’s effect on QRS widening may be mediated by inhibition of cellular gap junction communication which could explain the lack of effect of sodium bicarbonate described in case reports [3–6].

**Figure 1.**
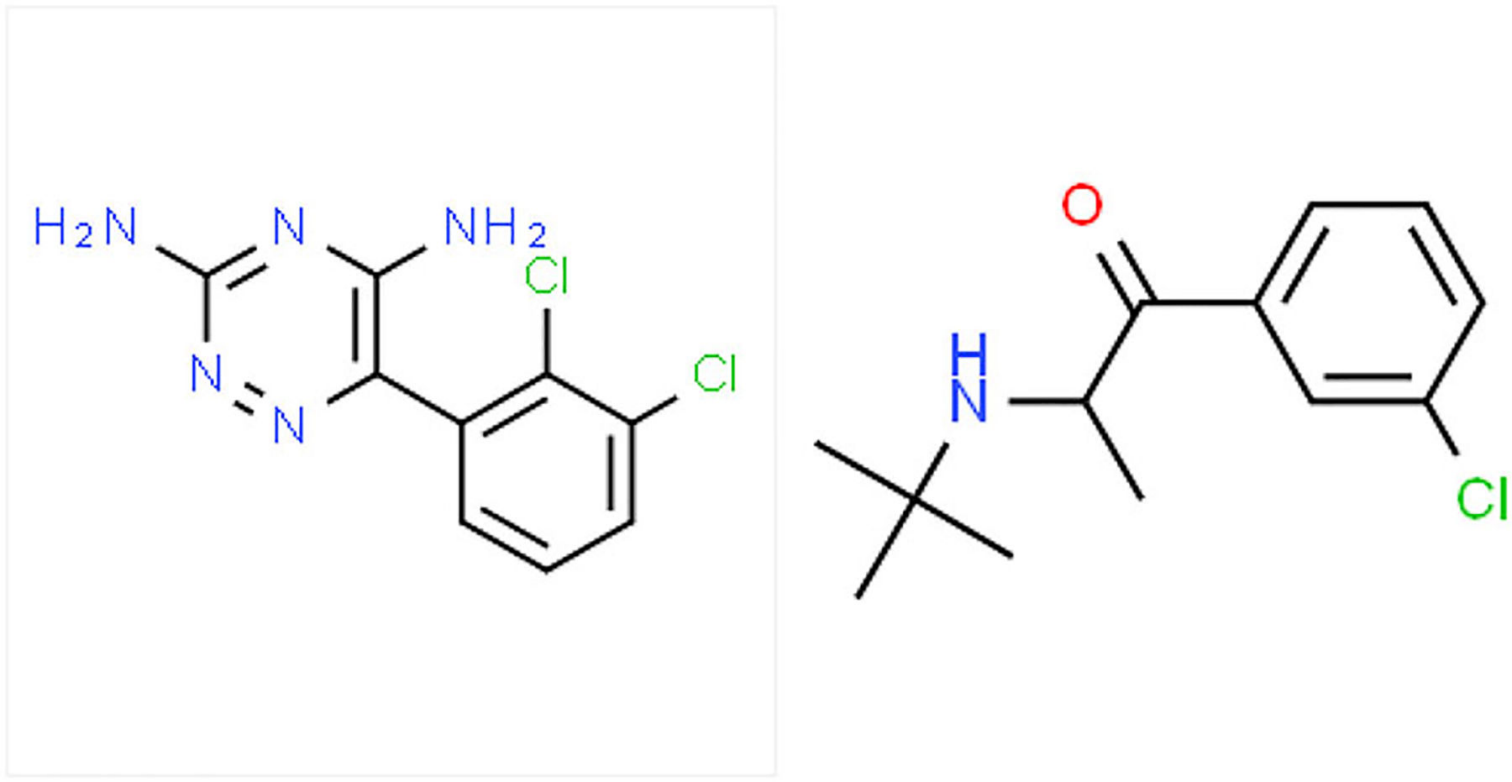
Lamotrigine (left) and Bupropion (right) Structures. Reference: CSID:3741, http://www.chemspider.com/Chemical-Structure.3741.html. (accessed 00:20, Aug 21, 2019), CSID:431, http://www.chemspider.com/Chemical-Structure.431.html. (accessed 01:04, Aug 21, 2019).

Lamotrigine is a phenyltriazine compound (Figure 1) anti-convulsant also used as a mood stabilizer. Please see Figure 1 for the structures of lamotrigine and bupropion. Its mechanism of action may be through inhibition of voltage-gated sodium channels. Its therapeutic range is 3–14 mcg/mL. It has a Vd of0.9–1.3 l/kg. In addition, it has no known active metabolites and an elimination half-life of 15–35 h.

Common toxic effects include rash, sedation, and nausea/vomiting. In overdose, QRS prolongation, dysrhythmias, coma, and seizures have been reported. Furthermore, similar to other anticonvulsants, lamotrigine can cause ataxia and nystagmus. It is also associated with hepatotoxicity. It may cause serotonin syndrome and QTc prolongation [7].

There have been multiple case reports of a serum lamotrigine concentration as high as 70–80 mcg/mL. For example, Chavez *et al* report a 36 y/o man with h/o HIV, bipolar disorder, benzodiazepine, alcohol, and cocaine abuse who took a massive lamotrigine overdose and had a lamotrigine concentration of 78.0 mcg/mL. His other medications besides lamotrigine were emtricitabine, raltegravir, and tenofovir. He had seizure activity with status epilepticus within one hour after presentation to the ED. He was treated with continuous veno-venous hemofiltration and 8.4% bicarbonate solution. He was also given lipid emulsion therapy after persistent QRS widening which resulted in normalization of the QRS interval. It is unclear what the timing of the peak concentration was in conjunction with administration of intralipid. An EEG 8 h after his presentation showed generalized seizures and he received phenobarbital [8]. Another case report involves a 26 year-old woman with severe menorrhagia after approximately 40 grams of lamotrigine. This patient’s peak lamotrigine concentration was 73 mcg/mL which was obtained on day 2 of hospitalization. She also exhibited more typical lamotrigine toxicity including nystagmus, ataxia, slurred speech, and seizures but she did not have QRS widening [9].

Sirianni *et al* described a 17 year-old girl with seizure activity, a widened QRS, and cardiovascular collapse which responded to lipid emulsion therapy [10]. At 6.5 h after ingestion and before lipid emulsion, her lamotrigine concentration was 26 mcg/mL, and her bupropion concentration was 180 ng/mL [10]. There is also another case of a 50-year-old woman who overdosed on lamotrigine and developed QRS widening that responded to lipid emulsion therapy but not sodium bicarbonate therapy. The highest concentration reported in this case report was 29.7 mcg/mL [11]. A 23 year-old patient in a case series had a concentration of 90 mcg/mL 16.7 h post ingestion. He presented with tonic-clonic seizures, agitation, and QRS widening. He received lipid emulsion therapy 60 h after ingestion [7].

In a review of acute lamotrigine overdose by Alyahya in 2018, 10 patients received sodium bicarbonate and they report 4 cases did not have QRS correction. Our case adds another case of a response to sodium bicarbonate [12]. Furthermore, this review also includes another case of reported bupropion and lamotrigine overdose resulting in status epilepticus approximately 10 h after initial EEG (it appears this was started as part of initial work up in her hospitalization). Her serum lamotrigine concentration was12.5 mcg/mL 12 h post admission to the hospital and timing of ingestion is unclear [12, 13].

This case illustrates that massive lamotrigine and bupropion ingestions can result in status epilepticus and QRS widening with high risk of cardiovascular collapse and require vigilant and attentive care.
